# The need to reassess single-cell RNA sequencing datasets: the importance of biological sample processing

**DOI:** 10.12688/f1000research.54864.2

**Published:** 2022-03-08

**Authors:** Alex M. Ascensión, Marcos J. Araúzo-Bravo, Ander Izeta

**Affiliations:** 1 Computational Biology and Systems Biomedicine Group, Biodonostia Health Research Institute, San Sebastian, Gipuzkoa, 20014, Spain; 2 Tissue Engineering Group, Biodonostia Health Research Institute, San Sebastian, Gipuzkoa, 20014, Spain; 3 Computational Biomedicine Data Analysis Platform, Biodonostia Health Research Institute, San Sebastian, Gipuzkoa, 20014, Spain; 4 IKERBASQUE, Basque Foundation for Science, Bilbao, Spain; 5 CIBER of Frailty and Healthy Aging (CIBERfes), Madrid, Spain; 6 Computational Biology and Bioinformatics Group, Max Planck Institute for Molecular Biomedicine, Münster, Germany; 7 Department of Biomedical Engineering and Science, Tecnun-University of Navarra, School of Engineering, San Sebastian, Gipuzkoa, 20009, Spain

**Keywords:** single-cell RNA-seq, skin, fibroblasts, reproducibility, computational analysis, Python

## Abstract

**Background:** The advent of single-cell RNA sequencing (scRNAseq) and additional single-cell omics technologies have provided scientists with unprecedented tools to explore biology at cellular resolution. However, reaching an appropriate number of good quality reads per cell and reasonable numbers of cells within each of the populations of interest are key to infer relevant conclusions about the underlying biology of the dataset. For these reasons, scRNAseq studies are constantly increasing the number of cells analysed and the granularity of the resultant transcriptomics analyses.

**Methods:** We aimed to identify previously described fibroblast subpopulations in healthy adult human skin by using the largest dataset published to date (528,253 sequenced cells) and an unsupervised population-matching algorithm.

**Results:** Our reanalysis of this landmark resource demonstrates that a substantial proportion of cell transcriptomic signatures may be biased by cellular stress and response to hypoxic conditions.

**Conclusions:** We postulate that careful design of experimental conditions is needed to avoid long processing times of biological samples. Additionally, computation of large datasets might undermine the extent of the analysis, possibly due to long processing times.

## Introduction

The quest for deciphering the underlying biology of numerous phenomena at the single-cell level has exponentially increased the number of published single-cell RNA sequencing (scRNAseq) studies. ^1^ Additionally, individual studies are gradually increasing in scale, and in most tissues a correlation between the numbers of cells sequenced and the number of identified cell types is found. ^1^ Unfortunately, many (if not most) of the studies concentrate their efforts on individual dataset analyses and perform relatively little correlative study to meta-analyse previously published scRNAseq datasets. However, the amount of information that could be retrieved from the already existing corpus of literature is enormous. ^2^

Within identified cell clusters (what we normally would define as ”cell types”), the existing cell heterogeneity may be indicative of cell subsets that respond to particular conditions (such as cell cycle phase, cell stress, response to local signals, etc.) or reflect underlying functional/positional differences. ^3- 6^ It is thus of utmost importance that the scientific community interested in a specific tissue or cell type agrees on the existing subsets within particular cell types and their defining molecular profiles, so that a common reference atlas may be used to understand homeostasis and response to varying insults. ^7^

In a re-analysis of 13,823 human adult dermal fibroblasts obtained from four independent scRNAseq studies, ^8- 11^ we recently proposed that human skin presents a common set of fibroblast subsets, irrespective of donor area. ^12^ These subsets can be categorised into three main fibroblast types (type A, B, and C), with a total of 10 minor subpopulations (A1–A4, B1–B2, C1–C4). In a recent landmark paper published in Science, Reynolds *et al*. produced a dataset of 528,253 sequenced cells obtained from healthy adult skin (five female patients undergoing mammoplasty surgery) and fetal samples, as well as inflamed skin from atopic dermatitis and psoriasis patients. ^13^ In healthy dermal fibroblasts, the authors described three populations: a main cluster termed Fb1, and two minor subpopulations, Fb2 and Fb3. Fb2 was additionally described as enriched in fetal and inflamed skin samples. ^13^ We aimed to analyse whether the Fb1, Fb2 and Fb3 populations were consistent with the A–C fibroblast types and subtypes that we had just described. More specifically, we reasoned that at least the most abundant subpopulations that we had defined, namely A1, A2, B1 and B2, should be clearly detected in a >500k cell dataset, thus further validating our previous scRNAseq study. In contrast, we found that a substantial proportion of the Reynolds *et al*. scRNAseq dataset shows a predominancy of differential expression of stress and hypoxia-related genes. Thus, data extracted from this source should be interpreted in the light of this bias. It is possible that other existing large datasets suffer from similar methodological problems, which might be due to insufficient oversight.

## Methods

### Preprocessing of fibroblast sample data

Fibroblast sample data originated from five donors, as described by Reynolds *et al*., ^13^ and were processed from raw fastq files (E-MATB-8142). The ID numbers are 4820STDY7388991 [S1], 4820STDY7388999 [S2], 4820STDY7389007 [S3], SKN8104899 [S4], SKN8105197 [S5]. Fastq files were processed using the loompy fromfq pipeline described in https://linnarssonlab.org/loompy/kallisto/index.html. *Loompy* (RRID:SCR_016666) and *kallisto* (RRID:SCR_016582) versions are 3.0.6 and 0.46.0. Genome fasta index and annotations were based on GRCh38 Gencode v31 (RRID:SCR_014966). Additionally, for other annotations and analysis of other populations, the processed h5ad adata from ^13^ was downloaded from the Zenodo repository (ID: 4536165).

Each individual sample (S1–C fibroblast types and subtypes that we had just descS5) data was processed equally using the following *scanpy* (RRID:SCR_018139, v1.7.0rc1) ^14^ procedure. To map the clusters from the original publication, cells from the processed data set were extracted and mapped to the samples. Genes with fewer than 30 counts were discarded. The sample was normalised ( sc.pp.normalize_per_cell) and log-transformed. Then, Principal Component Analysis (PCA) with 30 components was calculated and feature selection was performed with *triku* ^15^ (RRID:SCR_020977, v1.3.1), and *k*NN with cosine metric were computed. Finally, UMAP (RRID:SCR_018217, v0.4.6) ^16^ and leiden (v0.8.3) ^17^ were applied to detect the fibroblast populations.

Most of the cells from the preprocessed adata were mapped to the raw dataset. However, additional unmapped cells appeared, some of them related to other cell types (e.g. keratinocytes, immune cells or perivascular cells). To assign unmapped cells to their corresponding cell types a population matching algorithm was applied (described below). This algorithm requires a dictionary of cell types and markers. The markers used were the following:

• Fibroblast: *LUM*, *PDGFRA*, *COL1A1*, *SFRP2*, *CCL19.*
• Perivascular cell: *RGS5*, *MYL9*, *NDUFA4L2.*
• Erithrocyte: *HBB*, *HBA2*, *HBA1.*
• Immune cell: *TPSB2*, *TPSAB1*, *HLA-DRA*, *FCER1G*, *CD74.*
• Melanocyte: *PMEL*, *MLANA.*
• Endothelial vascular cell: *CLDN5*, *PECAM1.*
• Keratinocyte: *DMKN*, *KRT1*, *KRT5.*
• Mitochondrial content (low quality): *MTND2P8*, *MTND4P12*, *MTCO1P40*, *ADAM33*, *RN7SL2*, *MTRNR2L6.*

Once cell types have been assigned, non-fibroblast cells were discarded, and the PCA, triku, *k*NN, UMAP, leiden cycle was repeated to recalculate the new cell projection.

The sample S5 was discarded from the analysis due to its lack of *SFRP2* expression, a well established fibroblast marker that is expressed in the rest of samples. ^12^

Then, we separated the Fb2 population from the Fb1 and Fb3 populations for each dataset and applied the population matching algorithm to annotate them with the labels assigned from. ^12^ The genes used for the population assignation were the following:

• A1: *PI16*, *QPCT*, *SLPI*, *CCN5*, *CPE*, *CTHRC1*, *MFAP5*, *PCOLCE2*, *SCARA5*, *TSPAN8*
• A2: *APCDD1*, *COL18A1*, *COMP*, *NKD2*, *F13A1*, *HSPB3*, *LEPR*, *TGFBI*
• B1: *CXCL2*, *MYC*, *C7*, *SPSB1*, *ITM2A*
• B2: *SOCS3*, *CCL19*, *CD74*, *RARRES2*, *CCDC146*, *IGFBP3*, *TNFSF13B*
• C: *CRABP1*, *PLXDC1*, *RSPO4*, *ASPN*, *F2R*, *POSTN*, *TNN*

Next, all datasets with Fb1 and Fb3, or Fb2 populations were joined. We applied the previous processing routine and, to correct for batch effects, we used *bbknn* (v1.4.0) ^18^ with metric=angular and neighbors_within_batch=2 parameters.

To analyse the transcriptomic profile between Fb1 and Fb3, and Fb2 populations, we joined the two datasets and applied the same processing pipeline as before. We first characterised the genes driving the differences by obtaining the DEGs between the two sets of populations, and running GOEA with the first 150 DEGs of each category. The set of ontologies used was *GO Biological Process 2018* with the module *gseapy* (v0.10.4). ^19^ Then, to assess that the differences were due to cellular stress in the Fb2 population, we downloaded the lists of genes mentioned in the Results section (gene lists are available in the Github repository below), and genes appearing in more than two lists were selected. Then, the population matching algorithm was run against this list, and clusters with scores lower than 0.55 were assigned as ”Non-stress” clusters.

To analyse the differences in transcriptomic profiles within Fb1 and Fb3 populations, we obtained the DEGs between the two sets of A2 populations, which were the easiest to separate in clusters. By using that list of DEGs, we applied the population matching algorithm and divided the Fb1 and Fb3 populations into two halves. We then obtained the DEGs between the two halves and ran GOEA with the first 150 DEGs of each category, which revealed a hypoxia pattern in one of the halves. To assess that the differences were due to hypoxia, we downloaded the lists of hypoxia-related genes, and genes appearing in more than two lists were selected. Since some key genes (some glycolysis genes, or important genes appearing in one list) were missing, they were manually added to obtain a more robust list. Then, the population matching algorithm was run against this list, as well as the list of stress-related genes, and clusters with scores lower than 0.5 were assigned as ”Normal” clusters.

To replicate the analysis on the rest of the cell types, we used the processed h5ad file.

### Correction of stress and hypoxia cell states

In order to correct for stress and hypoxia cell states we used the sc.pp.regress_out implementation from *scanpy* on the stress and hypoxia scores. We first created two sub-datasets, one containing stress and normal cells, and another one with hypoxia and normal cells, and then the scores were regressed out. Finally, the common processing pipeline was applied. Additional correction methods can be seen in the notebooks in the Zenodo repository. ^20^

### Population matching algorithm

The aim of this algorithm is to assign a set of clusters to a set of labels, where each label contains a list of representative markers. For each label we extracted the matrix of counts of the genes belonging to the label. Then, we created a new matrix, where we assigned to each cell and gene the sum of the counts of the gene within its *k*NN, divided by the number of neighbours. This step reduced the noisiness of the expression, and also exacerbated the local expression of a gene and dampened the expression of sparse genes.

Gene expression values were substituted by the ranked index of their expression; and the values were divided by the largest index to sum 1. Therefore, the cell with the highest expression had a value of 1 for that gene, while the lowest expressed cell had a near 0 value. After this normalisation was applied to the rest of genes within the label, the mean of the normalised values across genes was computed, so that each cell had one value for that label.

After the previous steps were computed for the rest of labels, a new matrix with the number of clusters by the number of labels was computed. For each label and each cluster, the percentile of the normalised values within cells of that cluster was computed (percentile 70 by default). This helped reduce noise on normalised values, and assigned a unique number per cluster.

This algorithm allowed choosing for intermediate states, that is cell labels with a high similarity. By default, the label with the highest score per cluster was chosen. With the intermediate state option, labels that had a similar value as the label with the highest value were included. The difference in values was set as a threshold (0.05 by default), and labels with a difference of a value greater than the threshold were not merged.

## Results

### Reassessment of the main cell populations in a large skin dataset reveals the presence of clusters with stress- and hypoxia-related gene signatures

By using an unsupervised population-matching algorithm (details in processed notebooks available online ^20^) we observed that in each of the healthy donors analysed by Reynolds *et al*., ^13^ at least two independent fibroblast clusters expressed signature markers of the A1, A2, B1 and B2 populations. One set of cells corresponded to the Fb2 population, and the second set corresponded to the Fb1 and Fb3 populations. A joint analysis of all donors after batch effect correction showed that the cluster duplication observed in each individual donor could be replicated jointly. We therefore assumed that some global effect should be affecting the cells, i.e. Fb2 might be a copy of Fb1+Fb3 cells, although perhaps affected by some alteration. Differential gene expression (DEG) analysis between Fb2 and Fb1+Fb3 revealed an enrichment in ontology terms associated to cell stress (e.g. unfolded protein response, regulation of apoptotic process, mRNA catabolic process). We then designed a signature gene list composed of 50 DEGs commonly associated to stress in very different scRNAseq settings (e.g. *ATF3*, *BTG2*, *FOS*, *FOSB*, *GADD45B*, *HSPA1A/B*, *IER2/3*, *JUN*, *JUNB*, *NFKBIA*, *NR4A1/2*, *PPP1R15A*, *RHOB*). ^21- 25^ Using this signature, the Fb2 population over-expressed *BTG2*, *EGR1*, *FOSB*, *IER2*, *SOCS3*, and *ZFP36*, among others, indicating that these cells clustered together mainly due to cellular stress.

**Figure 1.**
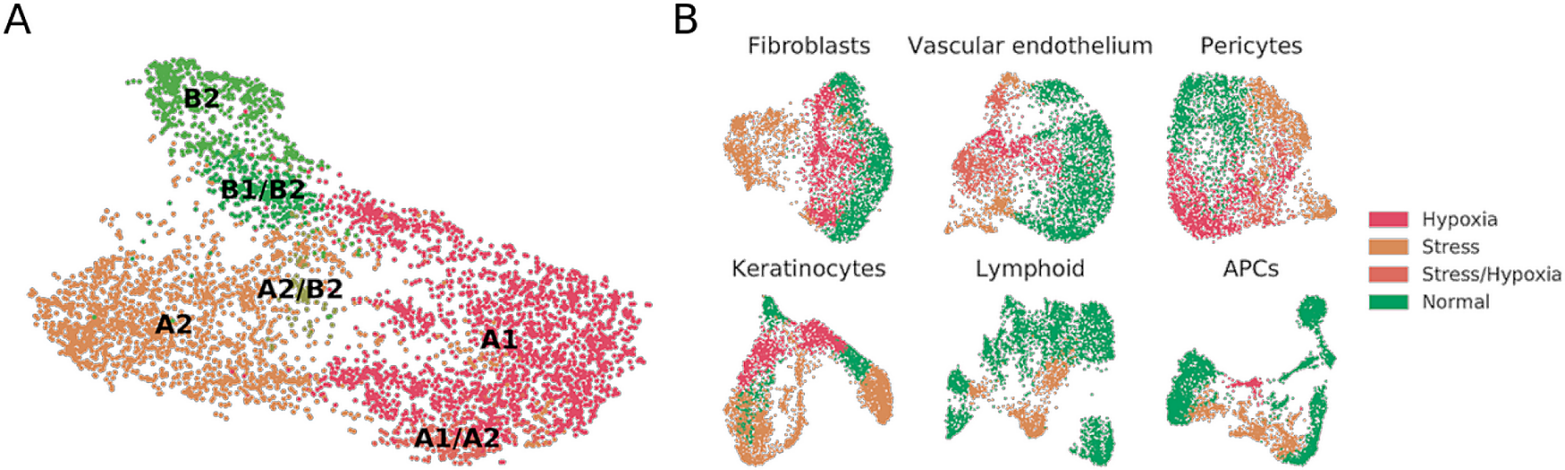
A re-analysis of the Reynolds et al. dataset in search of dermal fibroblast subpopulations reveals the presence of substantial proportions of stressed and hypoxic cells. (A) UMAP plot of normal fibroblasts (after removal of hypoxic and stressed cell subsets) reveals conservation of some, but not all, cell types previously described in independent datasets. ^12^ (B) UMAP plot of fibroblast, vascular endothelium, pericyte, keratinocyte, lymphoid and APC cell populations from healthy donors, labeled to highlight hypoxic and stressed cell subpopulations as characterized by overexpression of defined gene signatures.

In a further analysis of the Fb1 and Fb3 cells, we observed that the A1, A2, B1 and B2 populations appeared twice again. A DEG analysis between each pair of duplicated populations disclosed genes in one of the split populations that were related to glycolysis ( *ALDOC*, *ENO2*, *GAPDH*, *PGK1*, *PDK1*, *PFKFB4*, *PYGL*), cell integrity, hypoxia and apoptosis ( *BNIP3*, *BNIP3L*, *ANGPTL4*, *LOX*, *HILPDA*); whereas the second split population over-expressed units of the mitochondrial ATPase and complex I, indicating an active oxidative metabolism. It is well known that cells under hypoxic conditions switch from aerobic to anaerobic metabolism to keep energy homeostasis within the cell. ^26- 28^ We therefore generated a curated list of hypoxia-related genes, and managed to separate the non-hypoxic from the hypoxic group with the population-matching algorithm. Once stressed or hypoxic cells were removed on the basis of a set threshold of expression of signature genes, we mapped the main types of fibroblasts in what we termed normal cell subset of Reynolds *et al*. ( Figure 1A). Fibroblast A1, A2 and B2 populations were independently mapped, and we also found clusters which seemingly were mixtures of previously defined populations e.g. B1/B2, A1/A2, or A2/B2. No type C fibroblasts were detected.

To understand whether the stress and hypoxic signatures were only present in fibroblast subsets or could also be traced to other populations within the Reynolds *et al*. dataset, we mapped the stress and hypoxia gene signatures to perivascular cell, keratinocyte, vascular endothelial cell, lymphoid cell, and antigen presenting cell (APC) clusters. In our reanalysis of healthy donors, fibroblasts, perivascular cells, keratinocytes, and vascular endothelial cells showed clear hypoxia and stress-related clusters ( Figure 1B). For instance, the VE3 population, described by Reynolds *et al*. as increased in patients suffering from inflammatory conditions, presented a clear stress-related transcriptomic profile. On the other hand, most of the VE2 population over-expressed hypoxia-related genes. On lymphoid cells we did observe a sub-cluster of stressed Tc/Th cells but no clear hypoxic profiles. On APCs, an inflammatory macrophage cluster showed hypoxia, and the M2 and DC2 clusters showed stress-related profiles. Some of these results may be expected in physiological conditions for immune cells, but others could be attributed to sample handling.

**Figure 2.**
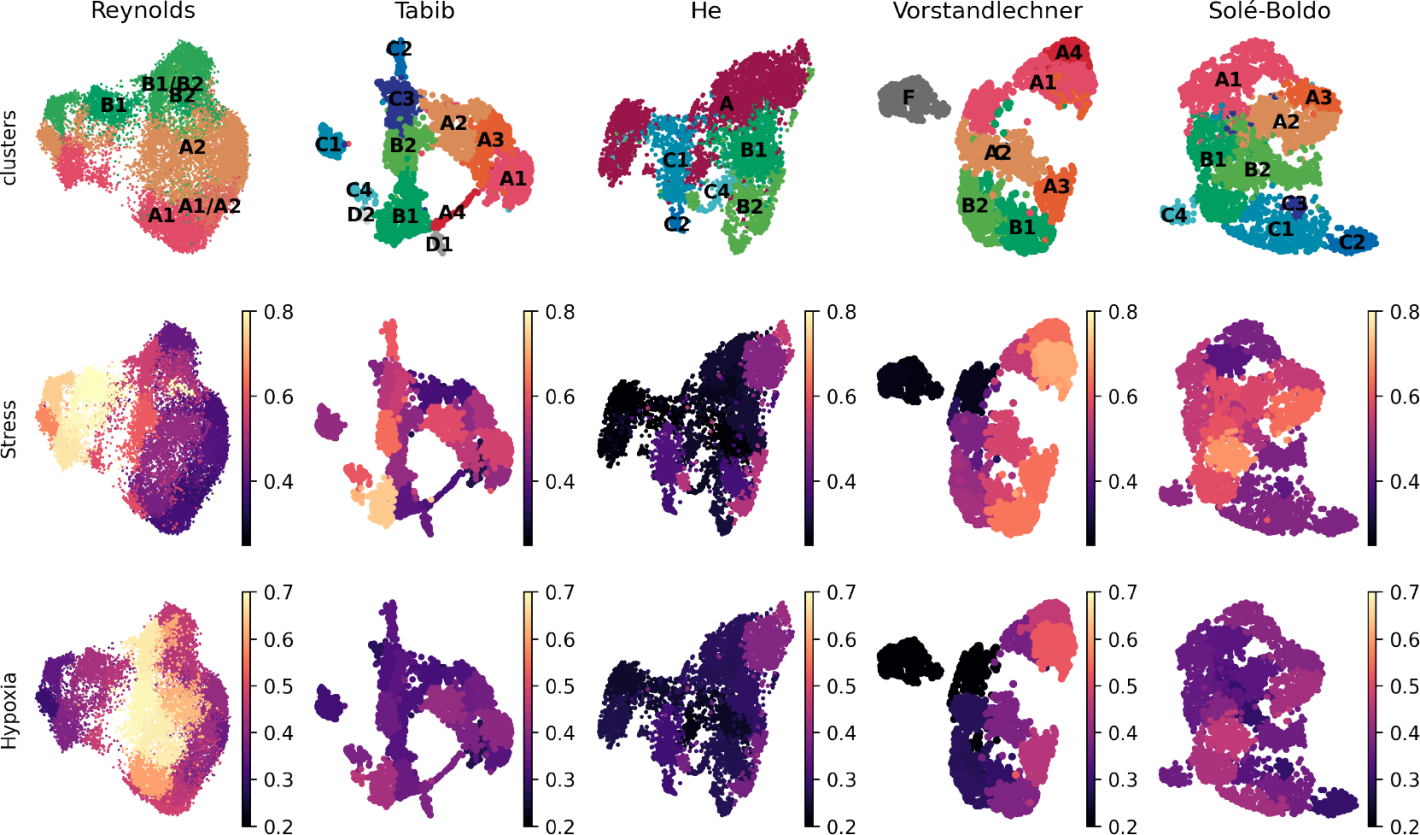
Stress and hypoxia-related signatures in published human dermal fibroblast datasets. (A) UMAP plot of normal fibroblasts (after removal of hypoxic and stressed cell subsets) reveals conservation of some, but not all, cell types previously described in independent datasets (1). (B) UMAP plot of human dermal fibroblast subsets as defined in ^12^ are shown here for five published datasets, ^8- 11, 13^ and depicted by the average levels of expression of stress and hypoxia gene signatures.

Finally, we tested if the aforementioned stress and hypoxia related signatures were present in the previously published scRNAseq datasets of human skin. ^8- 11^ The levels of expression of these genes were clearly higher in the Reynolds *et al*. dataset as compared to other available resources ( Figure 2).

### Correction of stress and hypoxia signatures shows that stressed cells show a non-recoverable gene signature

**Figure 3.**
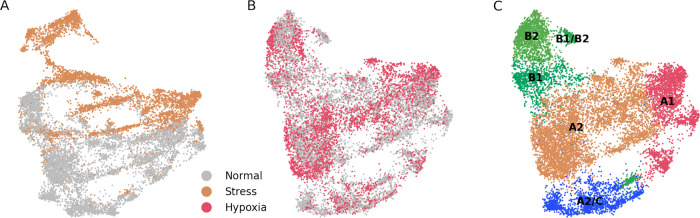
Dataset merging of stress and hypoxia populations show mixed degrees of integration with the ”normal” dataset. (A) UMAP plot of merged ”stress” and ”normal” cells. There is a low degree of integration between both cell types. (B) UMAP plot of merged ”hypoxia” and ”normal” cells. There is a high degree of integration between both cell types. (C) Unsupervised assignation of fibroblast types from (B) reveals, similar to results from Figure 1A, major fibroblast types.

Since the stress and hypoxia related expression profiles are apparent, we were interested in studying the ”reversibility” of the transcriptomic signatures, and creating a normalised dataset where hypoxic and stressed cells could merge with the normal cells, and classifying the whole dataset into the original cell types described in. ^12^ To this end, we applied two approaches with similar results. On the one hand, we considered cell states as batches, and applied batch effect correction with *bbknn* and *harmony.* On the other hand, we applied regression on the stress and hypoxia scores shown in Figure 2 based on the *Seurat*’s linear regression function implemented in *scanpy.* Since both approaches showed similar results, we show the results of the latter case in Figure 3.

To further study if stress and hypoxia transcriptomic profiles are ”recoverable”, we generated two types of datasets, one each with the stress or hypoxia cells, and another one containing normal cells. When applied the correction to the stress + normal dataset we observed that there was no integration between the two states ( Figure 3A). On the other hand, there was a good integration between the hypoxia and normal cell states ( Figure 3B), and the main fibroblast populations could be correctly mapped ( Figure 3C). From these results we infer that the transcriptome from stressed cells is much more altered than the one from hypoxic cells, to the extent that stressed cells are in a computationally non-reversible state.

### Analysis on running times of the Reynolds dataset with different numbers of cells

**Figure 4.**
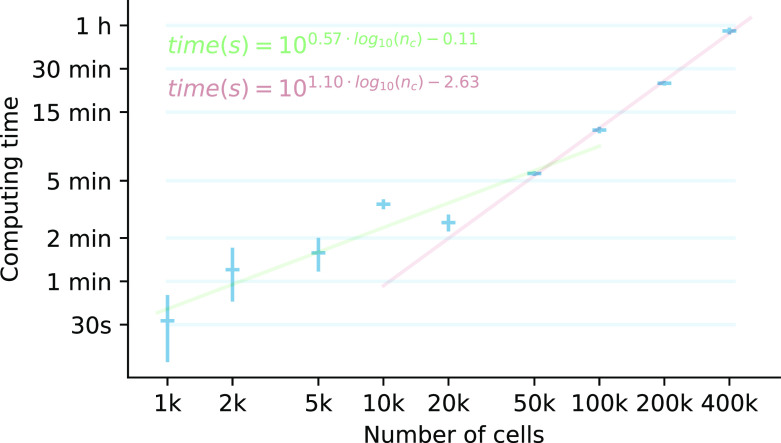
Running times of a basic single-cell pipeline on Reynolds dataset. For each number of cells, three running times are collected, and the mean (horizontal bar) and standard deviation (vertical bar) are shown. For the [1k - 20k] and [50k - 400k] intervals, two linear regressions were trained and extended outside of these intervals to show that there is a change in the processing time rate at ~30k cells. A doubling in the cell number implies 1.48 times more runtime in the [1k - 20k] interval, and 2.14 times in the [50k - 400k] interval.

The Reynolds dataset contains, after some basic filterings, approximately 450k cells. We became interested in analysing the runtimes of a standard single-cell pipeline procedure – consisting on quality control, PCA, graph neighbor construction, dimensionality reduction, clustering and DEG calculation – using different cell numbers, to see how this analysis is scaled. The results of the analysis are observed in Figure 4 and detailed in Supplementary Table 1.

The analysis shows that running the pipeline a single time in the whole dataset in a working station takes approximately 1 hour. The parts with the longest running times are the batch, clustering and DEG calculation. Additionally, when analysing trends in the processing times, we observe an inflection point at around 30,000 cells, marking two clear runtime trends. For higher numbers of cells, the processing times are further increased – 2.1 times per doubling of cells – compared with a lower number of cells – 1.5 times per doubling of cells.

For good measure, a single run of this pipeline analysis on a extended dataset with 1M cells would take 2 hours and 25 minutes.

## Discussion

The results from the efforts to compare, correlate, and compile the information present in available scRNAseq datasets could be condemned to short longevity since they can be overpassed by new resources that appear almost on a daily basis. However, it is to be expected that, at some tipping point, robust cell types and subtypes will be fully defined for each tissue and organ. Then, new scRNAseq datasets will only add information on the transcriptomically defined cell states of each of the robustly defined cell subpopulations, in response to specific perturbations such as injury or disease.

Here, we aimed to validate results we had obtained with a few thousand cells with a large scRNAseq dataset including over half a million cells. Instead, we have found that clustering of this large dataset shows an apparent pattern of expression of stress and hypoxia-related genes. In our opinion, the origin of stress- and hypoxia-related signatures in healthy donor cells might be caused by two factors: a tough tissue processing that put the cells under stress and hypoxia, and an underdeveloped analysis caused by the long processing time derived from the high number of cells, which hindered the detection of bad quality cells and propagated this artefact downstream the analysis.

The first factor is related to the very exhaustive and complex protocol for cell isolation chosen by authors. The top 200 *μ*m-thick layer of the skin was cut with a dermatome, digested with dispase (1 h at 37°C) to separate dermal and epidermal layers. Both layers were digested in collagenase for 12 h at 37°C, cells were filtered and subjected to FACS sorting before library generation and sequencing. ^13^ While this strategy warrants high purity of the obtained cell populations, the long processing times (≥16 h) and the use warm dissociation for a long period might have significantly affected patterns of gene expression of relevant numbers of cells in this setting. In this sense, aiming to process large numbers of cells involves longer processing times. High processing times (even ≥ 60’) have previously been reported to generate significant transcriptomic alterations. ^21, 25^ and, in particular, warm dissociation is associated with stress response, ^29^ which is apparent in the transcriptomic profiles of part of the cells . An alternative to warm dissociation may be the use of cold-active psychrophilic proteases. ^30^

The second factor is related to the computational and analytic challenges of such a complex dataset. As it has been observed in Figure 4, runtime of analytic pipelines vastly increase with the numbner of cells. Thus, if the processing time is expected to be the same for a small dataset and a big dataset, due to the low time to perform a beginning-to-end analysis adapted to the current fast-paced times of publication, this leads to a more shallow exploration at the initial stages. Before a pipeline is run on a single-cell dataset, researchers usually have to spend some time doing an exploratory analysis, where they select the cutoff values for quality control, explore different batch effect removal methods, or tune the parameters for clustering, neighbour graph calculation, and other steps in the pipeline.

These decisions are made on the basis of the output of the differently-preprocessed versions on downstream analyses: how the datasets look on UMAP plots, how robust their DEGs are, etc. Usually, this part of the analysis requires several reruns of the same pipeline to find the best parameters and obtain an overall view of the limitations of the dataset and the general information elements that will be obtained from it. This means that single-cell pipelines are not a linear, but rather an iterative process where researchers have to make decisions based on the output of previous steps. As a consequence, if the results from initial stages of the analysis are overlooked and biases go unnoticed, these effects propagate downstream the pipeline – e.g. observing differences in healthy vs diseased samples, search for rare populations, pathway/ontology analysis, or RNA velocity analysis – and artefacts can be presented as genuine results, hindering the dissemination of quality results to the scientific community.

In conclusion, understanding skin fibroblast heterogeneity is of great relevance not only in skin homeostasis, but also in ageing ^11, 31^ and disease. ^32- 36^ We sincerely hope these reanalyses help further advance the field of single-cell transcriptomics of human skin. Further refinement of fibroblasts subsets and their identity-defining features will provide a fruitful framework for the advancement of knowledge as well as for the development of novel therapeutic approaches in dermatological disease and skin cancer.

## Data availability

### Extended data

Repository: Extended data for “The need to reassess single-cell RNA sequencing datasets: the importance of biological sample processing”. https://doi.org/10.5281/zenodo.6324956. ^37^

This project contains the following underlying data:

Supplementary Table 1 (Processing times of different elements of the single-cell pipeline, varying the number of cells analysed).

Data are available under the terms of the license Creative Commons Attribution 4.0 International.

## Software availability

Notebooks to replicate this work can be found at: https://github.com/alexmascension/revisit_reynolds_fb.

Processed notebooks and AnnData files can be found at: https://doi.org/10.5281/zenodo.4596374. ^20^

License: Creative Commons Attribution 4.0 International.
