# Supplementary material for: The need to reassess single-cell RNA sequencing datasets: the importance of biological sample processing
Source: F1000Res. 2022 Mar 8;10:767. Originally published 2021 Aug 6. [Version 2] doi: 10.12688/f1000research.54864.2 (PMC8984215; doi:10.12688/f1000research.54864.2)
Supplement: Peer review history

# Peer review: Reviewer response for version 2

**DOI:** 10.5256/f1000research.122038.r126626

**Published:** 2022-04-05

**Authors:** Xiao Long, Zhujun Li

**Affiliations:** 1 Department of Plastic Surgery, Peking Union Medical College Hospital, Chinese Academy of Medical Sciences, Beijing, China

The authors made improvements according to the reviewers' comments. The revised title is better than the previous version.

Is the work clearly and accurately presented and does it cite the current literature?

Yes

If applicable, is the statistical analysis and its interpretation appropriate?

Yes

Are all the source data underlying the results available to ensure full reproducibility?

Yes

Is the study design appropriate and is the work technically sound?

Yes

Are the conclusions drawn adequately supported by the results?

Partly

Are sufficient details of methods and analysis provided to allow replication by others?

Yes

Reviewer Expertise:

Adipose derived stem cells

We confirm that we have read this submission and believe that we have an appropriate level of expertise to confirm that it is of an acceptable scientific standard.

# Peer review: Reviewer response for version 2

**DOI:** 10.5256/f1000research.122038.r126627

**Published:** 2022-03-22

**Authors:** (Jo) Huiqing Zhou

**Affiliations:** 1 Department of Molecular Developmental Biology, Faculty of Science, Radboud Institute for Molecular Life Sciences, Radboud University, Nijmegen, The Netherlands; 2 Human Genetics, Radboudumc, Nijmegen, The Netherlands

I am fully satisfied with the additions and comments that the authors have put together.

Is the work clearly and accurately presented and does it cite the current literature?

Yes

If applicable, is the statistical analysis and its interpretation appropriate?

Yes

Are all the source data underlying the results available to ensure full reproducibility?

Yes

Is the study design appropriate and is the work technically sound?

Yes

Are the conclusions drawn adequately supported by the results?

Partly

Are sufficient details of methods and analysis provided to allow replication by others?

Yes

Reviewer Expertise:

(Epidermal) stem cells; transcriptomis, epigenomics, developmental disease

I confirm that I have read this submission and believe that I have an appropriate level of expertise to confirm that it is of an acceptable scientific standard.

# Peer review: Reviewer response for version 1

**DOI:** 10.5256/f1000research.58386.r95141

**Published:** 2021-12-20

**Authors:** (Jo) Huiqing Zhou

**Affiliations:** 1 Department of Molecular Developmental Biology, Faculty of Science, Radboud Institute for Molecular Life Sciences, Radboud University, Nijmegen, The Netherlands; 2 Human Genetics, Radboudumc, Nijmegen, The Netherlands

In this article, the authors re-analysed a large dataset published in a high-impact journal and compared the outcome of analyzing this dataset with several other previously published works. This effort is highly appreciated and should be encouraged in the field, to critically evaluate and make sense of published work, with the ultimate aim to understand biology.

 Specifically, the authors re-analysed the skin single-cell RNA-seq data published by Reynolds *et al*., 2021. They initially focused on the healthy fibroblast population, using the authors’ previously published annotations, and identified an enhanced signature of stress- and hypoxia-related genes. They then further analysed several other cell populations of the dataset and reported that this enhanced stress and hypoxia signature is also present in other cell populations. Furthermore, using a normalised dataset where hypoxia and stressed cells could merge with the normal cells, they showed that the stress signature seems to be ‘irreversible’, in contrast to the hypoxia signature.

 The workflow and methods seem to be appropriate, and the conclusion on the enhanced stress and hypoxia gene signature in the analysed dataset is also convincing (for a non-computational biologist who has a good understanding of common bioinformatics analysis and interpretation). This is consistent with the authors’ discussions on the cell dissociation/processing methods. However, the title ‘more is not always better’ can be discussed and reconsidered. The authors should probably give advice on careful dissociation methods when retrieving a large number of cells, rather than proposing that a large number of cells is not necessary or desirable. In addition, the authors did not give an extensive discussion on data analysis effort in analyzing the large datasets. It is also appropriate for the authors to comment on the data retrieval process of the analyses dataset, and to encourage the authors of the original paper to annotate their data properly and clearly (e.g., which methods were used to generate which batch of data). This is true for all publications and it is the only way for scientists to share and re-use the published data according to the FAIR principle ( https://www.go-fair.org/fair-principles/).

Is the work clearly and accurately presented and does it cite the current literature?

Yes

If applicable, is the statistical analysis and its interpretation appropriate?

Yes

Are all the source data underlying the results available to ensure full reproducibility?

Yes

Is the study design appropriate and is the work technically sound?

Yes

Are the conclusions drawn adequately supported by the results?

Partly

Are sufficient details of methods and analysis provided to allow replication by others?

Yes

Reviewer Expertise:

(Epidermal) stem cells; transcriptomis, epigenomics, developmental disease

I confirm that I have read this submission and believe that I have an appropriate level of expertise to confirm that it is of an acceptable scientific standard, however I have significant reservations, as outlined above.

# Response

**Published:** 2022-03-02

**Authors:** Ander Izeta

**Affiliations:** Biodonostia Health Research Institute, Spain

We thank the reviewer for the helpful and insightful comments. We will now comment on the issues point-by-point.

*The title ‘more is not always better’ can be discussed and reconsidered*

 This comment is also in line with the other reviewer. Therefore we have changed the title to focus the paper mainly on biological sample processing.

*The authors should  probably  give  advice  on  careful  dissociation  methods  when  retrieving  a  large  number of  cells.*

 We agree with this excellent point. The original article had already quoted the reference by Denisenko *et al *(2020) where the importance of cold dissociation was demonstrated. The use of cold active psychrophilic proteases has been proposed to avoid use of enzymes that need incubation at 37ºC (Potter and Potter, 2019). Both references are now included in the text.

*[...] the authors did not give an extensive discussion on data analysis effort in analyzing the large dataset*

 We analyzed the dadaw- Source code can be located as scripts in the GitHub repository https://github.com/haniffalab/HCA\_skin as well as in the Zenodo repository https://zenodo.org/record/4249674. Although the scripts from the GitHub repository are conveniently ordered and are legible, the Zenodo repository does not include the output results and intermediate figures from the scripts, so we were unable to check the values and intermediate results. Also, for some parts of the scripts, further commenting would have been appreciated. Additionally, we observed that the file structure of the GitHub and Zenodo repository were not comparable. For instance, the \textit{Pipeline} folder with the structured analysis is lacking in the Zenodo repository, although some of its scripts are scattered through different folders. Another issue from the GitHub repository is that analysis scripts were uploaded in their final form to the repository (commits 1766cd, 9b520f, 9a361e and 123b7d, 8 Dec 2020). Considering that scripts from Zenodo were uploaded on November, some efforts to tidy the GitHub repository were made afterwards. However, a quick look at the scripts from the different sections shows a lack of variable consistency and file I/O, which implies a lack of reproducibility on their scripts. Additionally, despite a succinct explanation of the README file of the GitHub repository, the lack of commentaries on the scripts and the apparition of entire scripts that are not reflected in the methods difficult any replication effort.

 Regarding the initial analysis of the dataset, from the python scripts we observed a set of common thresholds for all batches ( *n\_genes $<$ 6000, n\_genes $>$ 400, n\_counts $>$ 1000, percent\_mito $<$ 0.2*). Although this is common practice, maybe a thresholding per batch would have been more convenient. For instance, we observed that sample SKN8105197 does not provide enough consistency in fibroblast marker expression. For convenience, this sample was removed from the analysis.

 After QC and feature selection, bbknn is run with default parameters, although in other notebooks harmony has also been partially used. bbknn seems to be favored as the reference batch correction method. The lack of commit history does not allow us to look for previous attempts with other methods.

 In the Pipeline/02 folder we found some scripts that use a logistic regression model for label stability prediction. We do not observe this script in the methods section.

 In the Pipeline/03 folder we observe that an enhanced reclustering was made with hand-picked DEGs which, according to the authors, favored the separation of clusters. Interestingly, some of these DEGs are found on the hypoxia/stress lists (ZFP36, HSPA1A, HSPA1B, DNAJB1, JUNB, ATF3, SOCS3, GADD45B, FOS), and others are ribosomal protein associated genes (RPL22, RPL37, RPL34). In our opinion, the selected DEGs might bias the reclustering for the segregation of hypoxic and stress populations.

*It is also appropriate for the authors to comment on the data retrieval process of the analyses dataset, and to encourage the authors of the original paper to annotate their data properly and clearly*

 Regarding the data retrieval process, neither the GitHub nor the Zenodo repositories host the code for data retrieval and preprocessing described in materials and methods.

# Peer review: Reviewer response for version 1

**DOI:** 10.5256/f1000research.58386.r98248

**Published:** 2021-12-01

**Authors:** Xiao Long, Zhujun Li

**Affiliations:** 1 Department of Plastic Surgery, Peking Union Medical College Hospital, Chinese Academy of Medical Sciences, Beijing, China

The study reanalyzed data from several single-cell RNA sequencing resources, proposing an interesting point that a larger number of cells could lead to compromised results. It is well designed and conducted. Bioinformatic and statistic methods were valid.

 The authors brought up an interesting point of view. However, large numbers of cells might not have a definite positive correlation with longer processing time. For example, as the authors discussed, the choice of the method during tissue processing could be an important contributing factor to the biased results. Therefore, the conclusion should focus more on the bias caused by processing time and choice of method, and maybe quality control measures, instead of larger cell numbers, because that would require further experiments and analysis to rule out these confounding factors.

Is the work clearly and accurately presented and does it cite the current literature?

Yes

If applicable, is the statistical analysis and its interpretation appropriate?

Yes

Are all the source data underlying the results available to ensure full reproducibility?

Yes

Is the study design appropriate and is the work technically sound?

Yes

Are the conclusions drawn adequately supported by the results?

Partly

Are sufficient details of methods and analysis provided to allow replication by others?

Yes

Reviewer Expertise:

Adipose derived stem cells

We confirm that we have read this submission and believe that we have an appropriate level of expertise to confirm that it is of an acceptable scientific standard, however we have significant reservations, as outlined above.

# Response

**Published:** 2022-03-02

**Authors:** Ander Izeta

**Affiliations:** Biodonostia Health Research Institute, Spain

We thank the reviewer for the insight on focusing more on the biological processing of the sample. Although we are limited by the information from the article, we believe that a poor execution of the sample processing is a leading factor inducing the artifacts from the analysis. Indeed, we added some references supporting the claim that a general stress transcriptomic profile is produced by the long times that cells endured warm dissociation.

 However, we believe that the high processing time and its probable consequence of insufficient analytical insight at the dataset cannot be ignored. In fact, we added a new figure (Figure 4) showing a relevant increase in running times when increasing number of cells analyzed. Additionally, We have observed a trend in publications at top-ranked journals that consist of the presentation of datasets with a diverse and complex set of cells, but which lack an in-depth analysis and do not produce biological results with enough insights. This trend affects, in the end, the quality of the datasets. In our opinion, this might be related to the limited time data scientists have been able to iterate analyses with that dataset.

 We thus postulate that prospective authors should revise sample processing strategies as well as data analysis protocols, so that sampling errors can be pinpointed and corrected upstream in the analysis pipeline. In conclusion, we have reformulated our statements to increment the importance of biological sample processing as suggested by the reviewer.
